# Cyst of right hepatic duct in children

**DOI:** 10.1093/gastro/goaf079

**Published:** 2025-09-18

**Authors:** Yuchen He, Duote Cai, Shuhao Zhang, Kun Zhu, Yi Jin, Qingjiang Chen, Zhigang Gao

**Affiliations:** Department of General Surgery, Children’s Hospital, Zhejiang University School of Medicine, National Clinical Research Center for Child Health, Hangzhou, Zhejiang, P. R. China; Department of General Surgery, Children’s Hospital, Zhejiang University School of Medicine, National Clinical Research Center for Child Health, Hangzhou, Zhejiang, P. R. China; Department of General Surgery, Children’s Hospital, Zhejiang University School of Medicine, National Clinical Research Center for Child Health, Hangzhou, Zhejiang, P. R. China; Department of Pathology, Children’s Hospital, Zhejiang University School of Medicine, National Clinical Research Center for Child Health, Hangzhou, Zhejiang, P. R. China; Department of General Surgery, Children’s Hospital, Zhejiang University School of Medicine, National Clinical Research Center for Child Health, Hangzhou, Zhejiang, P. R. China; Department of General Surgery, Children’s Hospital, Zhejiang University School of Medicine, National Clinical Research Center for Child Health, Hangzhou, Zhejiang, P. R. China; Department of General Surgery, Children’s Hospital, Zhejiang University School of Medicine, National Clinical Research Center for Child Health, Hangzhou, Zhejiang, P. R. China

## Introduction

Choledochal cysts (CCs) are cystic dilatations of the intrahepatic and/or extrahepatic biliary ducts that affect primarily female infants [1–3]. CCs vary markedly in incidence worldwide: the incidence is approximately 1 in 100,000 to 1 in 150,000 live births in Western countries and 1 in 1,000 people in Asian countries [4].

CCs are classified based primarily on the system proposed by Alonso-Lej and later refined by Todani *et al.* [5, 6]. According to Todani’s classification, type I CC, which involves dilatation of the common bile duct, is the most common. Type II involves diverticular dilatation; type III is characterized by dilatation of the inner segment of the duodenal wall of the common bile duct; type IV involves multiple dilations of the bile duct; and type V is defined as dilatation of one or several segments of the intrahepatic bile ducts [7]. Herein, we present three cases of pediatric CCs that did not fit the categories by Torani.

## Case report

### Case 1

A 5-year-old girl was admitted with recurrent paroxysmal periumbilical abdominal pain persisting for more than 3 years. Physical examination indicated a flat and soft abdomen with mild tenderness around the umbilicus. Her laboratory tests were normal.

**Figure 1.**
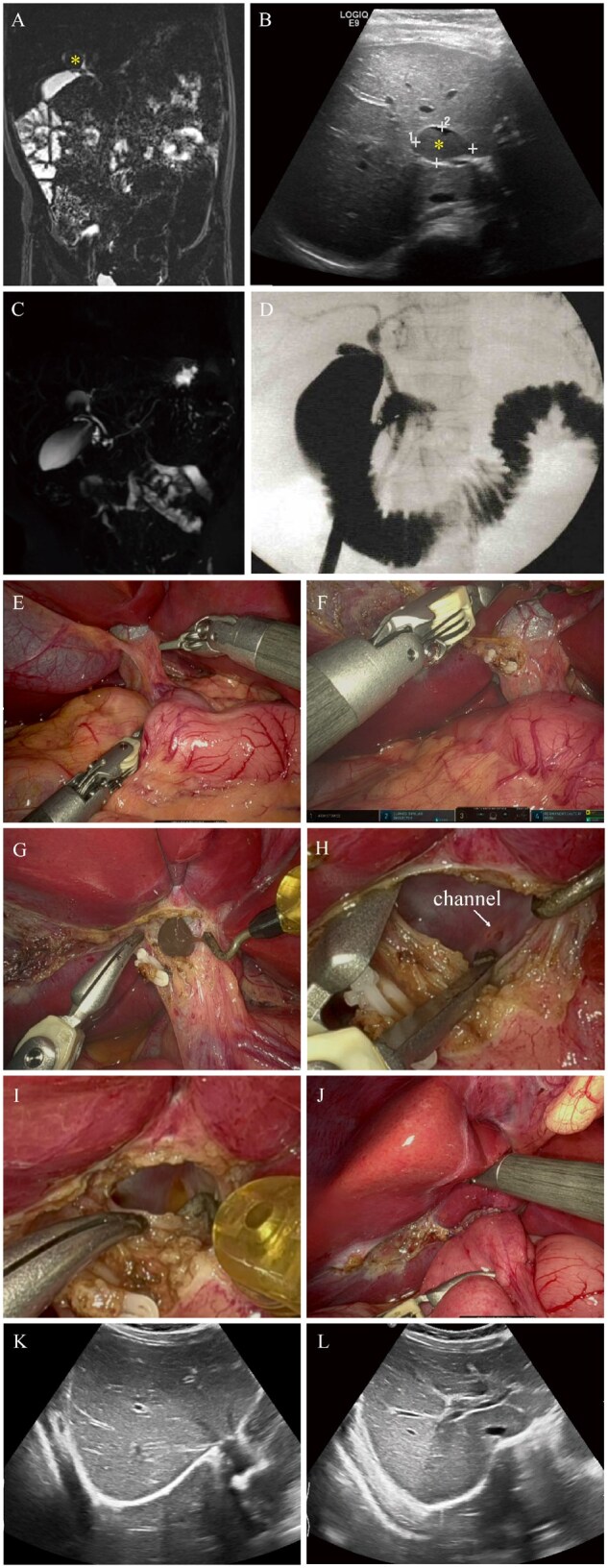
Imaging studies (abdominal ultrasound and MRCP) and intraoperative photographs of case 1. (Α) MRCP before surgery, indicating a round lesion near the hepatic portal vein, measuring approximately 1.9 cm × 1.4 cm and characterized by short T1 and long T2 signals. (Β) Abdominal ultrasound before surgery, indicating a cystic echo near the intrahepatic portal vein, which had increased in size to 2.1 cm × 2.1 cm × 1.3 cm. (C) MRCP before surgery. (D) Cholangiogram of the biliary system in case 1. (E) Comprehensive view of the porta hepatis. (F) Excision of the gallbladder. (G) Discharge of a viscous brownish fluid after opening of the cyst. (H) Channel revealed by puncture and enlargement of the cyst. (I) Bile-like fluid flowing slowly from the channel into the cyst cavity. (J) Anastomosis of the jejunal biliary branch of a right hepatic duct cyst. (K and L) Abdominal ultrasound after surgery, indicating no significant dilation of the intrahepatic or extrahepatic bile ducts.*: cyst.

An abdominal ultrasound conducted on 16 November 2022, revealed a 1.9 cm × 1.8 cm × 1.1 cm cystic lesion, which was located in front of the intrahepatic portal vein. Magnetic resonance cholangiopancreatography (MRCP) indicated a round lesion, measuring 1.9 cm × 1.4 cm, near the hepatic portal system (Figure 1A). An abdominal ultrasound conducted on 26 May 2024, revealed a cystic echo near the intrahepatic portal vein; its size of 2.1 cm × 2.1 cm × 1.3 cm was greater than that observed in the prior abdominal ultrasound. The cyst maintained clear boundaries and showed the presence of bile sludge (Figure 1B), as confirmed through MRCP (Figure 1C).

Given the patient’s pain and her family’s request for surgical intervention, she underwent Da Vinci-assisted cholangiography, cholecystectomy, and jejunal Roux-en-Y anastomosis (Figure 1D). Her gallbladder was removed, thus exposing the right hepatic duct cyst (Figure 1E and F). After cystotomy, a viscous brown liquid was released (Figure 1G). The cystic cavity was then thoroughly rinsed, and the cyst incision was expanded. We identified a channel inside the cyst (Figure 1H), which contained a small amount of golden, bile-like fluid (Figure 1I). The liquid that drained from the cyst was tested, which revealed nearly undetectable amylase. A biliary anastomosis to the right hepatic duct cyst was performed (Figure 1J; Supplementary Video 1).

Postoperatively, the patient’s abdominal pain resolved without complications. Postoperative ultrasonography revealed no significant dilation of bile ducts (Figure 1K and L).

### Case 2

A 5-year-old girl was admitted with an abdominal mass that had been present for 5 years and upper-right-quadrant pain that had been present for 2 years. Her physical examination findings revealed a soft, non-tender abdomen. Her laboratory tests yielded normal findings.

An initial abdominal ultrasound conducted on 18 August 2016, revealed a cystic area measuring 2.0 cm × 2.0 cm × 1.8 cm (Supplementary Figure 1A). Regular examinations indicated that the cyst had remained stable. During hospitalization, an abdominal ultrasound conducted on 16 September 2021, revealed a 3.4 cm × 2.4 cm cyst at the confluence of the left and right hepatic ducts (Supplementary Figure 1B), and MRCP revealed a diverticular connection to the right hepatic duct (Supplementary Figure 1C).

Because she was experiencing repeated pain, we performed Da Vinci-assisted cholecystectomy and Roux-en-Y anastomosis of the cyst to the jejunum. Cholangiography confirmed the presence of a cyst with dimensions of 3 cm × 2 cm in the right hepatic duct (Supplementary Figure 1D). The surgical procedure began with a dissection of the portal vein (Supplementary Figure 1E and F). The gallbladder was isolated and removed, allowing for better exposure of the cyst within the porta hepatis (Supplementary Figure 1G). The cyst was punctured to examine its contents, and a bile-like fluid was drained (Supplementary Figure 1H). Subsequently, the opening of the right hepatic duct cyst was enlarged. The internal wall of the cyst exhibited structural similarities to a normal bile duct (Supplementary Figure 1I). No protein plugs or substantial bile outflow were observed. Amylase testing of the cyst fluid revealed 1.9 U/L. After thoroughly rinsing the cystic cavity, we performed cystic jejunal biliary branch anastomosis (Supplementary Figure 1J). Postoperatively, the patient’s abdominal pain resolved without complications.

Annual follow-up indicated that the cyst remained stable, with dimensions of approximately 1.5 cm × 1.3 cm × 1.0 cm, and minor internal stones were present (Supplementary Figure 1K). Proper drainage was confirmed (Supplementary Figure 1L).

### Case 3

A 9-year-old girl was admitted with an abdominal mass that had been present for 9 years. She had been admitted to another hospital for diarrhea, and an abdominal ultrasound identified a liver cyst. After treatment, her condition improved, and she attended follow-up evaluations. One year prior, a re-examination at another hospital prompted concerns regarding the cyst’s hepatic origin. A recent physical examination had revealed that her abdomen was flat and soft, and laboratory tests indicated normal levels.

After admission to our center, an abdominal ultrasound conducted on 21 August 2023, indicated cystic dilatation of the common bile duct, with dimensions of 3.5 cm × 3.3 cm × 1.7 cm. A thick wall and hypoechoic deposits measuring 1.8 cm × 0.6 cm were observed (Supplementary Figure 2A and B). MRCP conducted on 30 August 2023, revealed a common bile duct diameter of 4 mm, with an irregular shadow in the porta hepatis, measuring 3.4 cm × 1.7 cm, which was poorly demarcated with respect to the right hepatic duct (Supplementary Figure 2C and D). A subsequent abdominal ultrasound conducted on 2 July 2024, revealed cystic dilatation of the common hepatic and right hepatic ducts, with dimensions of 3.5 cm × 2.7 cm × 2.0 cm; and hypoechoic deposits measuring 2.0 cm × 0.6 cm inside the cyst (Supplementary Figure 2E and F).

Given the success of the first two surgeries, the patient underwent Da Vinci-assisted cholangiography, cholecystectomy, and jejunal Roux-en-Y anastomosis. Cholangiography confirmed patent right and left hepatic ducts, and the right hepatic duct cyst was not clearly visible (Supplementary Figure 2G). The gallbladder was removed. After cystotomy, a cloudy, yellow fluid was drained, and viscous stones were discharged (Supplementary Figure 3A–D). Incision of the cyst wall revealed a smooth inner surface (Supplementary Figure 3E). A suspected channel was discovered; however, no apparent bile flow was observed after flushing (Supplementary Figure 3F). A right hepatic duct cyst jejunal Roux-Y anastomosis was performed. Postoperatively, the abdominal pain resolved without complications, and the cyst tissue was subjected to pathological sectioning and immunohistochemical staining analysis (Supplementary Figure 4).

## Discussion

Numerous atypical cases of CCs that do not fit the Todani classification have been documented in the recent literature. The diagram of Todani classification and the right hepatic duct is provided in Supplementary Figure 5. In 1996, a 45-year-old patient with a hepatic duct cyst who underwent cystectomy and left hepatic duct jejunostomy was reported [8]. In 2023, Miron *et al.* reported a patient initially misdiagnosed with a type II Todani cyst. However, intraoperatively, the cyst was found at the hepatic duct confluence and showed a complex morphology that did not conform to Todani type [9].

To the best of our knowledge, this study is the first report of hepatic duct cysts comprising comprehensive case histories. The characteristics of hepatic duct cysts can be summarized as follows:

1. The cysts are located at the right hepatic duct near the porta hepatis.
2. The cyst is similar to a diverticulum, with the proximal end connected to the hepatic duct or atresia, and the cyst wall has channel-like remnants.
3. Normal hepatic duct morphology and alignment are not affected by the cyst.
4. The cyst contains bile or sludge entering through the connecting duct and is free of amylase. If the duct is blocked, the cystic fluid becomes non-biliary.
5. The histopathology of the cyst wall is concordant with bile duct tissue.
6. Liver function is normal.
7. The presence of right upper abdominal pain is variable and may be mild.

In summary, hepatic duct cysts in children are rare. At present, no definite conclusion has been reached regarding the optimal surgical approach for hepatic duct cysts. At our center, we prefer to use cholecystectomy and jejunal Roux-en-Y anastomosis as the surgical treatment modality. Whether malignant transformation occurs after surgery and the prognosis still require us to conduct longer-term follow-up for further observation.

## Supplementary Material

goaf079_Supplementary_Data
