# Supplementary material for: Cyst of right hepatic duct in children
Source: Gastroenterol Rep (Oxf). 2025 Sep 18;13:goaf079. doi: 10.1093/gastro/goaf079 (PMC12449143; doi:10.1093/gastro/goaf079)
Supplement: goaf079_Supplementary_Data [file goaf079_supplementary_data.zip › Supplementary Data.docx]

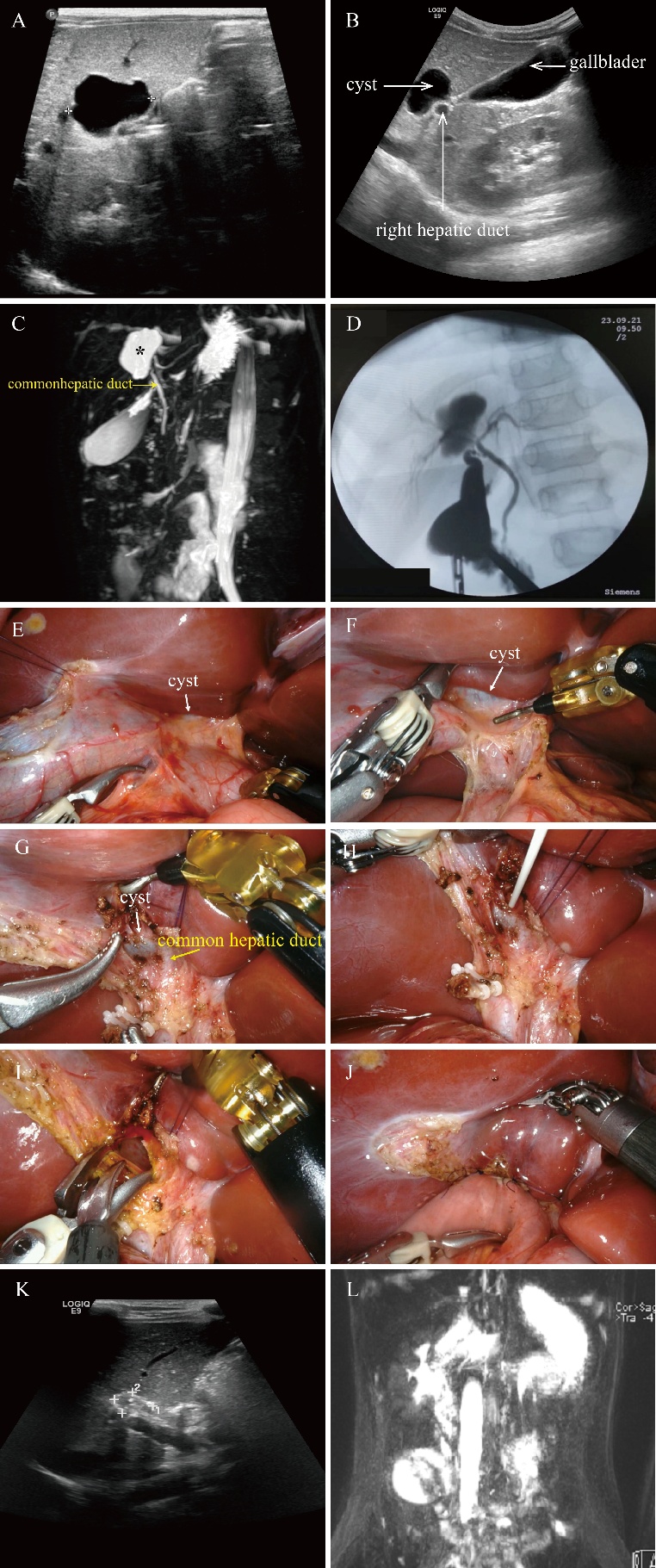


**Supplementary Figure 1.** Imaging studies (abdominal ultrasound and MRCP) and intraoperative photographs of case 2. (A) Initial abdominal ultrasound, indicating a cystic area measuring 2.0 cm × 2.0 cm × 1.8 cm, with low echogenicity. (Β) Abdominal ultrasound before surgery, indicating a 3.4 cm × 2.4 cm cyst at the junction of the left and right hepatic ducts. (**C**) MRCP before surgery, indicating a diverticular connection to the right hepatic duct, without main duct dilation. (D) Cholangiogram of the biliary system in case 2. (E) Comprehensive visual representation of the porta hepatis. (F) Anatomy revealing a right hepatic duct cyst. (F) Circumferential dissection of the CC. (G) Excision of the gallbladder. (H) Puncture of the cyst. (I) Inner wall of the cyst, exhibiting a structure similar to that of a normal bile duct wall. (J) Anastomosis of a right hepatic duct cyst with a biliary branch of the jejunum. (K) Abdominal ultrasound after surgery, indicating that the cyst remained stable, at approximately 1.5 cm × 1.3 cm × 1.0 cm, and contained minor internal stones. (L) MRCP after surgery.


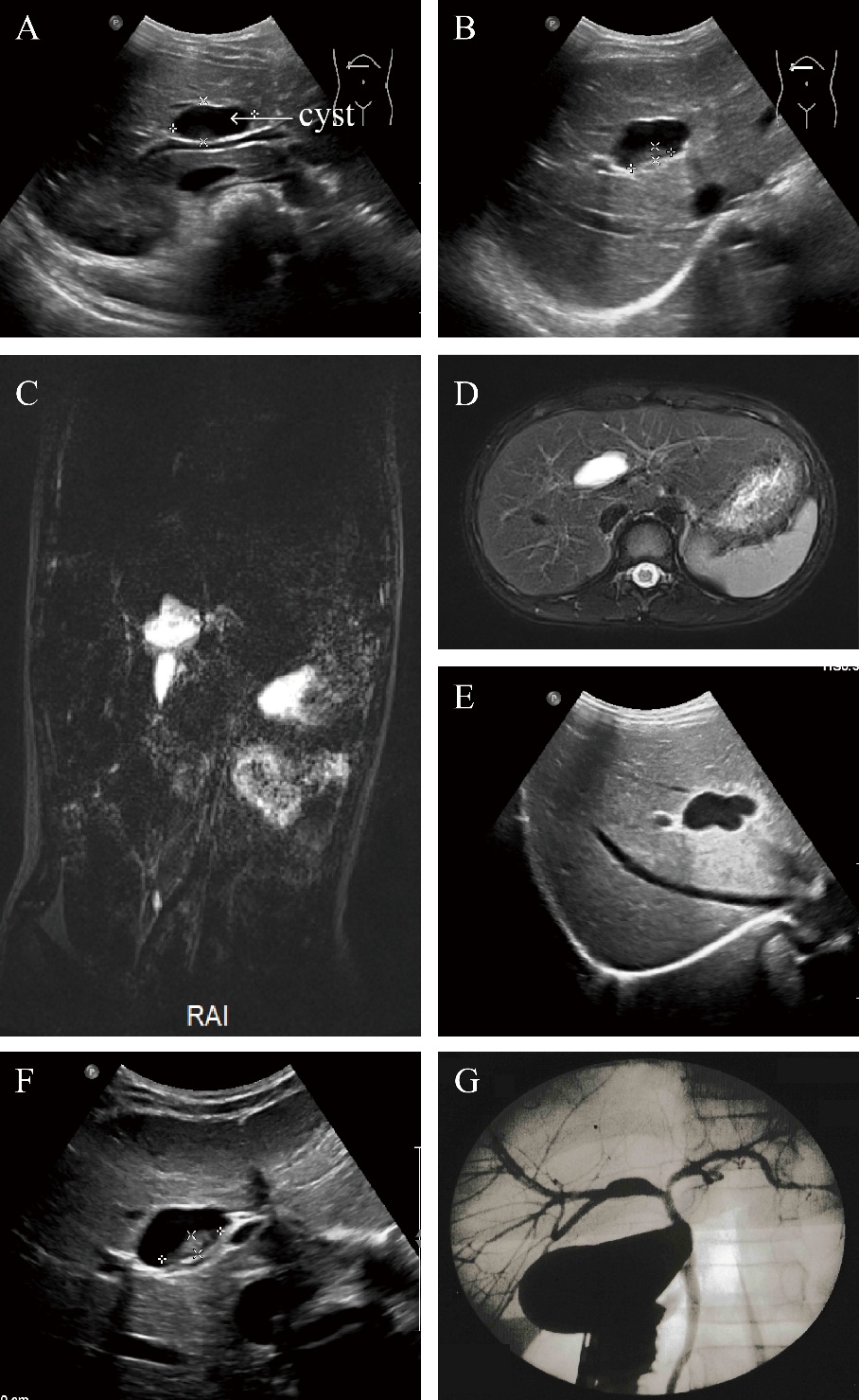


**Supplementary Figure 2.** Imaging studies (abdominal ultrasound and MRCP) of case 3. (Α, Β) Abdominal ultrasound before surgery, showing a cystic dilatation of the common bile duct, measuring 3.5 cm × 3.3 cm × 1.7 cm, with a thick wall and hypoechoic deposits measuring 1.8 cm × 0.6 cm. (C, D) MRCP before surgery, indicating a common bile duct diameter of approximately 4 mm, with an irregular long T1 and T2 signal shadow in the porta hepatis. (E, F) Abdominal ultrasound before surgery, indicating a cystic dilatation of the common hepatic and right hepatic ducts, measuring 3.5 cm × 2.7 cm × 2.0 cm, and containing internal hypoechoic deposits measuring 2.0 cm × 0.6 cm. (G) Cholangiogram of the biliary system.


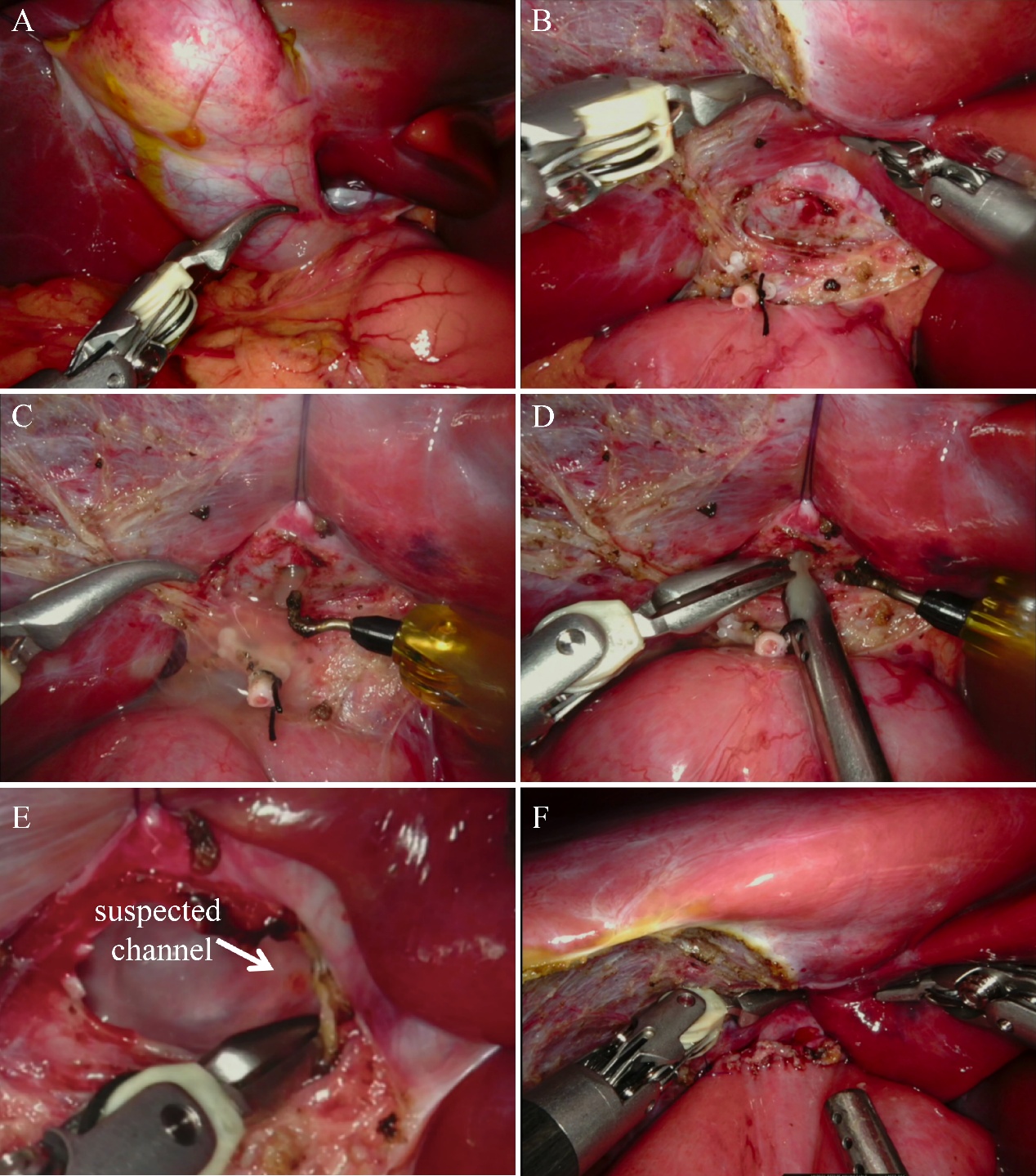


**Supplementary Figure 3.** Intraoperative photographs of case 3. (A) Full view of the porta hepatis. (B) Removal of the gallbladder. (C) Opening of the cyst and discharge of a cloudy, yellowish fluid. (D) Discharge of a viscous, unformed, sediment-like stone from the cyst. (E) The inner wall of the capsule. (F) Anastomosis of the jejunal biliary branch of a right hepatic duct cyst.


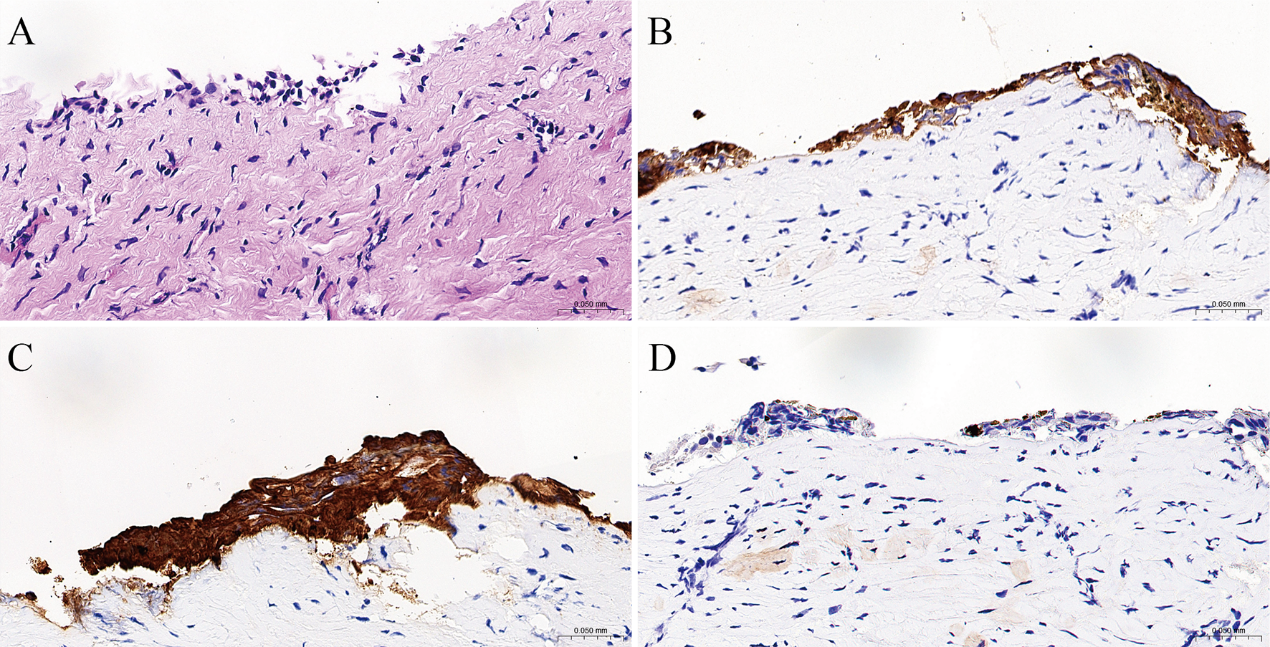


**Supplementary Figure 4.** Pathology sections from case 3. (A) Hematoxylin-eosin staining. (B) Cytokeratin 7 positive. (C) Cytokeratin 19 positive. (D) Cytokeratin 20 negative.


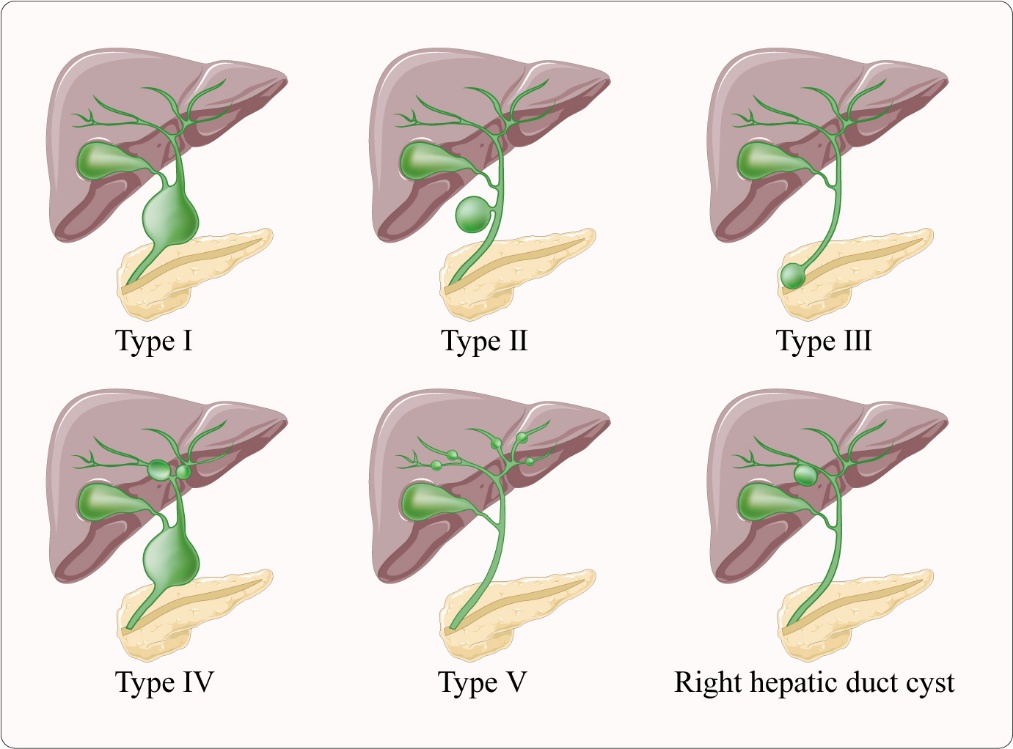


**Supplementary Figure 5.** Todani classification of common bile duct cysts and schematic diagram of the cyst location in the right hepatic duct.
